# Towards optimizing the service pathway “back to work” for people with acquired brain injury

**DOI:** 10.3389/frhs.2026.1754914

**Published:** 2026-07-06

**Authors:** Katarzyna Karcz, Priska Fritsche, Thomas Geisen, Yvonne Keller, Benno Muff, Gabriella Riemer-Kafka, Frank Staudenmann, Barbara Zimmermann-Gerster, Monika E. Finger

**Affiliations:** 1 Swiss Paraplegic Research, Work and Integration Group, Nottwil, Switzerland; 2 FRAGILE Suisse Patient Organization, Zürich, Switzerland; 3 Institute for Integration and Participation, University of Applied Sciences for Social Work North-Western Switzerland, Olten, Switzerland; 4 Disability Office Lucerne, Integration Department, Lucerne, Switzerland; 5 Social Security and Labour Law, Professor Emerita, University of Lucerne, Lucerne, Switzerland; 6 Rehabilitation Clinic Bellikon, Work-orientated Rehabilitation Bellikon, Switzerland; 7 Employers’ Association, Social Policy Department, Zürich, Switzerland

**Keywords:** acquired brain injury, integrated knowledge translation, RTW service pathway, sustainable work, vocational integration

## Abstract

**Introduction:**

Rising costs and increasing complexity place growing pressure on healthcare and social security systems worldwide. For people with acquired brain injury (ABI), successful return to work (RTW) depends not only on medical recovery but also on timely, coordinated support across healthcare, social security, vocational integration, and employment systems. Although Switzerland provides comprehensive healthcare and social protection, services relevant to RTW remain highly fragmented, posing substantial challenges for sustainable employment after ABI.

**Objective:**

This study aimed (1) to develop a system-level understanding of current RTW service pathways for people with ABI in Switzerland, with particular attention to interactions, discontinuities, and coordination gaps across sectors, and (2) to describe key features of an optimized, lifelong RTW service pathway as a reference for planning and coordinating services to support sustainable employment across the working life.

**Materials and methods:**

The study applied an Integrated Knowledge Translation (IKT) approach, involving researchers, people with ABI, representatives of patient organizations, healthcare professionals, vocational integration specialists, employers' representatives, and social insurance stakeholders as active project partners. Following a three-stage process—information gathering, mapping of current (“as-is”) service pathways, and identification of the key features of an optimized pathway—we combined evidence from prior qualitative research with newly collected data from online surveys of Swiss neurorehabilitation clinics and cantonal disability insurance offices.

**Results:**

Current RTW service pathways for people with ABI were mapped, revealing substantial fragmentation across settings and actors. Key gaps were identified and grouped into four interrelated themes: information, coordination, communication, and financing. These gaps affect multiple transition points and limit continuity of support over time. Through iterative discussion and refinement within the IKT framework, core elements of an optimized RTW service pathway were identified to support individuals with ABI, professionals, and employers in facilitating sustainable work participation.

**Conclusions:**

A pathway-oriented perspective highlights how fragmented services and coordination failures undermine sustainable employment after ABI. This study identifies critical gaps in current RTW service pathways and outlines both short-term practice-oriented measures and longer-term structural and policy-level directions for improvement. Actively involving stakeholders across sectors enhances the relevance and feasibility of pathway-level recommendations and increases the likelihood of their implementation in practice.

## 1 Introduction

Return to work (RTW) is a central outcome for people with acquired brain injury (ABI) and a key determinant of long-term psychosocial wellbeing, economic independence, and social participation (1). Successfully resuming or maintaining employment after ABI, however, rarely depends on medical recovery alone. Instead, it unfolds along a complex service pathway that spans healthcare, rehabilitation, vocational integration, social security, and the workplace itself (2, 3). Breakdowns or delays anywhere along this pathway can endanger sustainable employment, yet how these services interact over time remains poorly understood in many health and welfare systems (4).

ABI encompasses all brain injuries occurring after birth, including traumatic brain injury (TBI) and stroke (5). It can result in persistent physical, cognitive, emotional, and behavioral impairments that substantially affect daily functioning and work capacity (6, 7). Increasing evidence conceptualizes ABI as a chronic condition (8), requiring coordinated and often lifelong access to healthcare, vocational, and psychosocial services (9, 10).

The population affected is substantial and growing. TBI has been described as a “silent epidemic” due to limited societal awareness of its long-term consequences, while stroke incidence among people under 50 years has increased in Europe since the 1990s. Globally, an estimated 73 million working-age people live with ABI, including approximately 90,000 in Switzerland (11, 12). Even after so-called mild or minimal TBI, around half of individuals may experience long-term cognitive impairments that interfere with work performance and employment sustainability (13).

Returning to work after ABI is strongly associated with improved quality of life, identity reconstruction, and mental health (14–16) while also reducing the economic burden on individuals, their relatives and employers (17). Yet employment outcomes remain highly variable: international RTW rates for people with ABI range between 35% and 71%. In Switzerland, systematic national data on employment rates, RTW trajectories and service utilization are lacking. Available evidence—largely derived from patient organizations and qualitative studies—points to substantial difficulties in achieving and maintaining employment, but these findings remain fragmented and insufficiently connected to a system-level understanding of RTW-relevant services and transitions (18–20).

Work, a central dimension of social participation, is particularly critical for people with disabilities, including those with ABI (21, 22). Compared to the general population, people with disabilities face greater difficulties in finding and maintaining employment due to health-related limitations and systemic barriers (16, 23, 24). Reduced labour market participation also increases reliance on disability benefits while diminishing social capital and tax revenue (25).

In parallel, Switzerland—like other high income countries—faces increasing pressure on health and social security systems due to rising costs and demographic change (26). These challenges underline the importance of RTW strategies that extend beyond medical recovery to address long-term employability and sustainable work participation. As a result, policy strategies promoting coordination across sectoral boundaries—often framed as integrated care—have therefore gained prominence (27, 28). For working-age populations, such strategies increasingly emphasize not only health outcomes but also social participation, including gainful employment. However, services relevant to RTW frequently remain fragmented and uncoordinated across healthcare, social security, vocational rehabilitation, and labour market systems.

In Switzerland, these coordination challenges are particularly pronounced due to the highly differentiated institutional context in which RTW after ABI takes place. Individuals at risk of economic hardship or social exclusion due to illness or accident are protected by a comprehensive but complex social security system involving disability insurance, accident insurance, occupational pension insurance, and supplementary benefits (25, 29). Healthcare financing differs fundamentally between illnesses and accidents, responsibilities are distributed across multiple insurers and cantonal authorities, and vocational reintegration is primarily governed by disability insurance (DI).

ABI rehabilitation in Switzerland typically follows two main phases: medical neurorehabilitation and vocational rehabilitation (30). Medical neurorehabilitation delivered in inpatient and outpatient settings, focusses on therapeutic recovery and is funded by health or accident insurance. Vocational rehabilitation focuses on work capacity and job placement and is founded mainly by DI or accident insurance. It is delivered by DI staff or external specialists. Neurologists and rehabilitation physicians play a central gatekeeping role, as their assessment of diagnosis, functional limitations and work capacity strongly influences eligibility for vocational measures and benefits.

For people with ABI, whose successful RTW depends on the timely coordination of healthcare providers, insurers, vocational integration specialists, employers, and social services, this institutional complexity creates significant risks of discontinuity. Recent evidence indicates that coordination between healthcare and social security actors—particularly between rehabilitation services and DI—is often insufficient for this population (31). Divergent institutional mandates, legal requirements, and cantonal regulations further complicate communication, shared planning, and continuity across settings.

Although a growing international literature has examined RTW after ABI, most studies focus on individual predictors of employment or on isolated components of rehabilitation or compensation systems, often within more centralized welfare contexts. Established theoretical frameworks, such as Loisel's biopsychosocial model of work disability and the Krause disability prevention framework, highlight the importance of dynamic interactions between workers, workplaces, healthcare systems, and compensation structures (32–34). However, there are currently no empirical studies in Switzerland that examine these interactions in people with ABI longitudinally within its fragmented, multi-actor system.

Care pathways are widely used to structure healthcare delivery and implement evidence-based practices (35). However, these pathways typically remain confined to the healthcare sector. For people with ABI, whose RTW depends on coordinated interactions across healthcare, social security, vocational integration, and employment settings, such narrowly defined care pathways are insufficient. Effective service pathways “back to work” must therefore be conceptualized as a cross-sectoral, longitudinal service pathway that integrates medical rehabilitation, vocational integration, workplace accommodation, and employment maintenance over time.

Conceptualizing RTW after ABI in this way has methodological implications. Because pathways are shaped by interactions among multiple actors and institutions, no single stakeholder perspective can adequately capture their functioning or points of breakdown. Integrated Knowledge Translation (IKT) therefore provides a suitable framework, enabling the co-production of context-sensitive knowledge with people with ABI, professionals, insurers, and employer representatives (36).

Accordingly, this study is among the first to examine RTW after ABI in the Swiss context adopting a longitudinal, cross-sectoral service pathway perspective, with a focus on interactions across healthcare, social security, vocational integration, and employment-related settings. Specifically, the study aimed (1) to develop a system-level understanding of current RTW service pathways for people with ABI in Switzerland, with particular attention to interactions, transition points, and coordination gaps across sectors, and (2) to describe key features of an optimized, lifelong RTW service pathway as a reference for planning and coordinating services to support sustainable employment across the working life.

This study constitutes the first part of a larger project, that aims to develop stakeholder-specific recommendations and to formulate policy and labour-market proposals to improve structural conditions for sustainable employment of people with ABI across their life course, as a case in point.

## 2 Methods

### 2.1 Study design and methodological approach: integrated knowledge translation (IKT)

This study employs a mixed-methods, multi-stakeholder health systems research design, combining findings from two previously completed qualitative studies (19, 20, 37, 38), purpose-designed institutional surveys, and an IKT framework in which knowledge users participated actively throughout the research process (39). No new thematic analysis of raw qualitative data was conducted; the qualitative evidence drawn upon in this study derives from two prior projects described under Data sources. New empirical data were generated through the institutional survey and through the collaborative knowledge production process within the IKT framework.

The IKT approach was selected because describing a contextualized, coordinated service pathway for people with ABI requires the integration of perspectives from across all relevant systems. IKT is a model of knowledge co-production in which knowledge users work together with researchers as active participants throughout the entire research process (21, 27, 28), enabling problems, barriers, and potential solutions to be identified in the immediate context in which they occur. In this project, the IKT approach structured both the composition of the project team and the conduct of each research stage, with knowledge users involved from initial planning through to pathway formulation.

Knowledge users were invited during the project planning phase by the research team (MF, KK) on the basis of two explicit selection criteria: direct professional involvement in at least one phase of the work reintegration pathway for people with ABI, spanning acute and post-acute neurorehabilitation, vocational integration, social security law, disability insurance administration, employer representation, and professional education; and institutional affiliation enabling them to act as change agents within their respective sector. The team was designed to cover the full continuum from early neurorehabilitation to sustained employment and to represent the key systems whose coordination is central to the research question. The resulting team comprised a person with lived experience of ABI, a representative of the patient organization FRAGILE Suisse, a rehabilitation and insurance physician, a lawyer specializing in social security law, a vocational integration specialist with additional background in disability insurance, a representative of a cantonal disability insurance office, a researcher from the University of Applied Sciences and Arts Northwestern Switzerland with expertise in social work and integration, and a representative of the Swiss Employers' Association.

The process was led jointly by the researchers (MF, KK), who convened and facilitated all project team meetings. Over the course of the project, ten online meetings of approximately 90 min each were held at six-week intervals, with scheduling adapted to project milestones and the academic calendar. All meetings were conducted via video conference, which enabled consistent participation from knowledge users located across different cantons and institutional settings. Meeting agendas and slides were circulated before and after each session to ensure continuity and shared understanding across the team.

### 2.2 The development process

A three-stage approach was used to describe the crucial aspects of the optimal service pathway: 1) information gathering, 2) mapping of the as-is situation and 3) identification of the key features of an optimized, lifelong RTW service pathway.

#### 2.2.1 Information gathering

**Figure 1:**
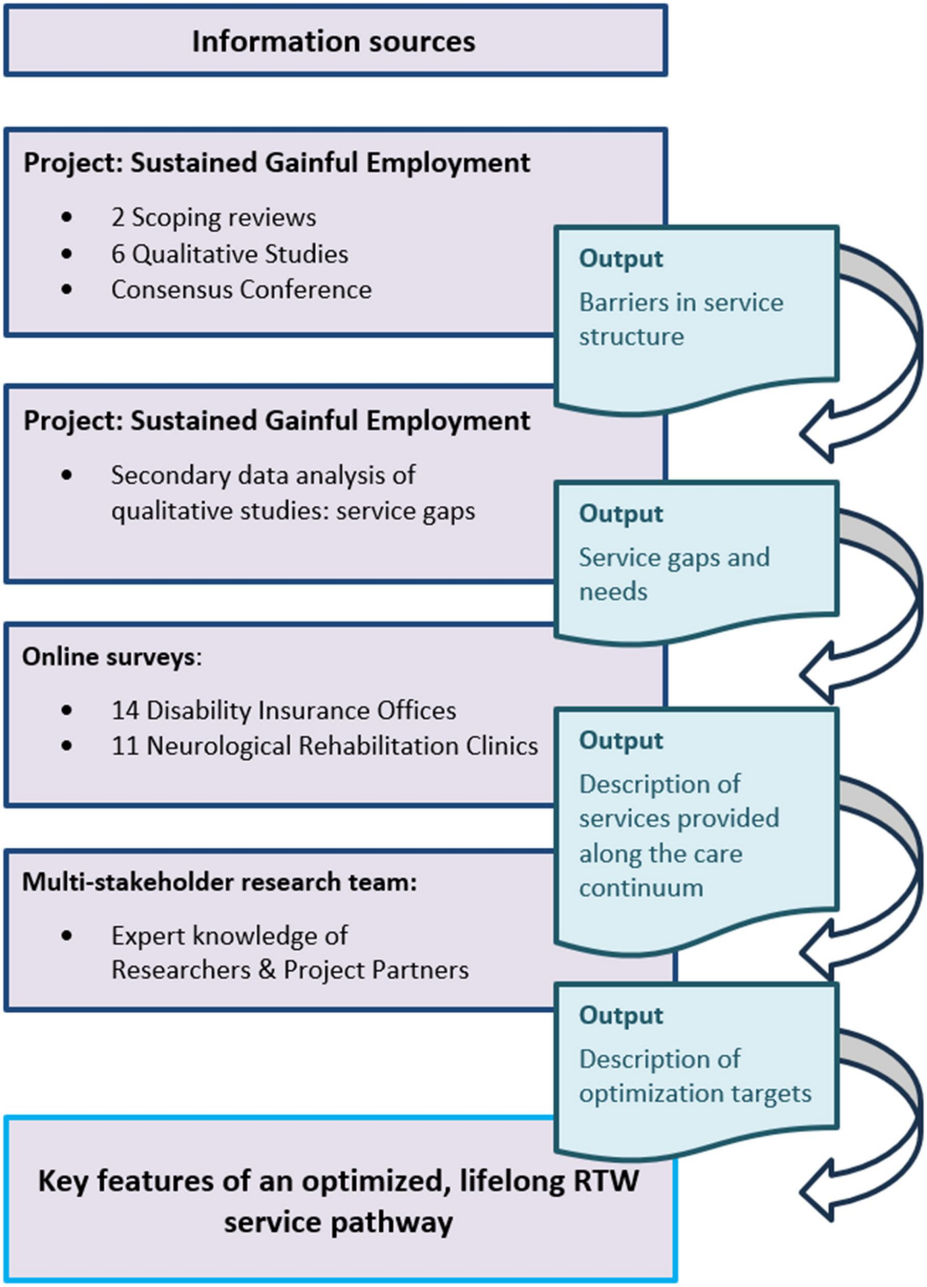
Information flow and how it was used in the study outcome.

To address our research question, we drew on multiple data sources (see Figure 1). Rather than re-analyzing raw qualitative data, we utilized findings from two previously completed studies—the Sustained Gainful Employment project and the subsequent secondary analysis of service gaps (31)—alongside two newly conducted institutional surveys of neurorehabilitation clinics and cantonal DI offices, and knowledge co-produced with project team members through the IKT process. Together these sources provided a multi-perspective empirical base covering the experiences of people with ABI, health and vocational professionals, employers, and institutional service providers, and formed the foundation for the analytical process described below.

#### Data source: Project “Sustained Gainful Employment”

The project identified barriers and facilitators to sustainable employment for individuals with ABI and spinal cord injuries (SCI) (40). Thereby we conducted focus group discussions and semi-structured interviews (41–43) with individuals with ABI and SCI (20), health and vocational integration professionals (19), and employers of persons with ABI and SCI (38). Data was collected between May 2019 and July 2020. An important finding was that the availability and coordination of medical and vocational integration measures and services across settings and sectors, specifically for people with ABI, were seen as a major obstacle to vocational integration and the sustainability of their employment. People with ABI also emphasized how structural fragmentation placed additional invisible demands on them, effectively shifting coordination work onto them and thereby impacting self-efficacy, fatigue, and employment sustainability.

To learn more about perceived service gaps and their relation to the identified risk factors for persons with ABI, we conducted an in-depth secondary analysis of the qualitative data (31).

#### Data source: service gaps paper

In a secondary analysis (31), the collected data were re-analyzed thematically (41, 42). While in the original project the perspective of all three stakeholders was analyzed separately and mainly risk factors for sustainable work in persons with ABI and SCI were compared, in the secondary analysis we combined the perspectives of the stakeholders and focused on risk factors and services impacting work integration in persons with ABI. Two major themes were identified: 1) person-related factors (including the subthemes: post-ABI impairments; lack of understanding of post-ABI impairments; poor health management) and 2) environment-related factors (including the subthemes: challenges related to the service structure; insufficient knowledge and education about ABI; challenges at the workplace; difficulties in private life). While stakeholders noted the variety of the currently available services, they particularly pointed to the missing long-term monitoring and counseling services for people with ABI following the initial RTW, reflecting a major challenge for sustainable work. An overarching gap related to the fragmentation of the service structure and the lack of case coordination along the working life.

#### Data source: online surveys

To map the availability and delivery of vocational integration services for people with ABI in Switzerland, two purpose-designed online surveys were conducted, combining closed-ended items (binary yes/no questions, multiple-choice ratings, and frequency scales) with open-text fields. Survey items were developed by the research team in consultation with project partners within the IKT framework. Both survey instruments were piloted prior to distribution: they were first reviewed by researchers familiar with the Swiss health and social security system and subsequently completed by project team members representing different stakeholder groups, who assessed comprehensibility, face validity, and practical usability; instruments were refined based on feedback from both rounds.

#### Survey 1: neurorehabilitation clinics

The survey was directed at all neurorehabilitation clinics in the German speaking part of Switzerland affiliated with SWISS-Reha (44). Initial invitations were sent to clinic directors, who were asked to answer the survey themselves or forward it to the staff member responsible for vocational integration, however only three responded. In a second step, the persons responsible for questions regarding professional integration at the clinic were identified and contacted directly and asked to forward the invitation to the survey to experienced knowledge users within the clinic. The survey covered seven thematic areas: inclusion of vocational integration in rehabilitation planning; professional disciplines involved; availability of comprehensive work capacity assessment; integrated case management; vocational counseling; work-oriented therapeutic services (occupational therapy, neuropsychology, physical training); and measures directly linked to job re-entry (workplace assessment and adaptations, job coaching, vocational skills training, job placement support, socio-vocational rehabilitation, preparatory work placements). Availability was rated separately for inpatient and outpatient settings. A final block addressed insurer and employer contact and external referral practices at discharge.

#### Survey 2: cantonal disability insurance offices

The survey was distributed to all 20 German-speaking cantonal DI offices via the project's DI partner. Survey items followed the structure of the official Swiss DI intervention catalogue [KSBEM, version 1 July 2022 (45)]. The early intervention phase covered eight measure categories: workplace adaptations, assessment of workplace aids, training courses, job placement and workplace retention support, vocational counseling, socio-vocational rehabilitation, occupational measures, and counseling and support. The vocational integration phase covered 15 categories: case management, vocational-medical work capacity assessment, counseling and support, coaching, placement search, socio-vocational rehabilitation (build-up and work training), occupational bridging measures, vocational counseling, formal and other vocational training, job placement and job search support, workplace retention, trial employment, and personnel leasing. For each measure, respondents rated (a) service delivery structure (DI staff/external providers/case-dependent/shared); (b) perceived effectiveness (ineffective/quite effective/very effective); and (c) frequency of use (most cases/about half/rarely or never/don't know). A final block addressed ABI-specific staffing, case assignment practices, and staff training.

In the second stage, the current (“as-is”) service situation was mapped. Findings from all data sources were systematically analyzed by the research team and jointly discussed to develop a shared understanding of the existing service landscape. This process enabled the identification of where services were available, where they were absent or insufficiently coordinated, and where transitions between settings or systems posed challenges. The preliminary results were subsequently presented to the project team and further refined through iterative discussion and feedback.

#### 2.2.3. Identification of the key features of an optimized, lifelong RTW service pathway

Service gaps identified across all data sources were synthesized using a thematic approach. These gaps were grouped into four overarching themes—information, communication, coordination, and financing—which were confirmed by the project team as the domains most consistently associated with suboptimal RTW outcomes for people with ABI. These themes subsequently informed the identification of key features of an optimized, lifelong RTW service pathway.

## 3 Results

### 3.1 Survey of neurorehabilitation clinics

All 11 SWISS-Reha clinics participated (response rate: 100%). All confirmed that vocational integration is addressed in their clinic; however, only five (45%) systematically incorporated it into rehabilitation goal setting and planning, five did so on a case-by-case basis, and one did not at all. Multidisciplinary teams—typically including occupational therapists, neuropsychologists, social workers, and physicians—were responsible for vocational integration across all clinics. Dedicated vocational integration specialists, vocational counselors, or job coaches were available only in a minority (Table 1).

**Table 1:** Availability of vocational services in neurorehabilitation clinics (*N* = 11).

Vocational service	Inpatient only *n* (%)	Outpatient only *n* (%)	Both settings *n* (%)	Not offered *n* (%)	Available any setting *n* (%)
Work capacity assessment	1 (9%)	1 (9%)	5 (45%)	4 (36%)	7 (64%)
Integrated case management	1 (9%)	1 (9%)	1 (9%)	8 (73%)	3 (27%)
Vocational counseling	—	2 (18%)	1 (9%)	8 (73%)	3 (27%)
Employer contact	1 (9%)	—	8 (73%)	2 (18%)	9 (82%)
Work-oriented occupational therapy	1 (9%)	1 (9%)	5 (45%)	4 (36%)	7 (64%)
Work-oriented neuropsychology	1 (9%)	1 (9%)	5 (45%)	4 (36%)	7 (64%)
Work-oriented physical training	1 (9%)	1 (9%)	4 (36%)	5 (45%)	6 (55%)
Workplace assessment and adaptations	—	2 (18%)	2 (18%)	7 (64%)	4 (36%)
Assessment of workplace aids	2 (18%)	3 (27%)	3 (27%)	3 (27%)	8 (73%)
Job coaching at the workplace	—	4 (36%)	1 (9%)	6 (55%)	5 (45%)
Vocational skills training courses	—	1 (9%)	1 (9%)	9 (82%)	2 (18%)
Job placement support	—	2 (18%)	—	9 (82%)	2 (18%)
Socio-vocational rehabilitation	—	4 (36%)	—	7 (64%)	4 (36%)
Preparatory work placements/internships	1 (9%)	1 (9%)	—	9 (82%)	2 (18%)
Referral to external VR at discharge	9 (82%)	2 (18%)*	—	—	11 (100%)

VR, vocational rehabilitation. Percentages reflect the number of clinics offering the service in the stated setting(s) as a proportion of all 11 participating clinics. “—”, not applicable or zero.

* Referral at discharge: 9 clinics (82%) routinely refer; 2 (18%) do so on a case-by-case basis; none reported never referring.

#### 3.1.1 Availability of vocational services

Work-oriented occupational therapy, work-oriented neuropsychology, and work capacity assessment were each available in seven of 11 clinics (64%), predominantly across both inpatient and outpatient settings; work-oriented physical training was available in six (55%). The quality and timing of work capacity assessments was a concern: one respondent described their clinic's assessment as “*interdisciplinary, certainly not comprehensive, and often too early*”, and another noted that the process “*is an interdisciplinary process, often coordinated by outpatient social counseling*”, pointing to the central coordinating role of social work where it was available. Four clinics (36%) offered no structured work capacity assessment at all.

Services closer to actual job re-entry were far less common and mostly confined to outpatient settings. Workplace assessments and adaptations were offered by four clinics (36%), described where available as occurring “*partly in direct collaboration with the DI or accident insurance*”. Job coaching at the workplace was available in five clinics (45%), provided “*selectively, as needed, by occupational therapy or social counseling*”. Vocational skills training, job placement support, and preparatory work placements were each available in no more than two clinics (18%). One clinic illustrated how insurance funding boundaries constrain even active vocational programs, noting that socio-vocational rehabilitation was provided “*only within the framework of regular medical therapy, with no DI-financed training offered*”.

#### 3.1.2 Case management and vocational counseling

Integrated case management—defined as structured coordination involving the person with ABI, the medical team, insurers, and employer—was available in only three clinics (27%); eight (73%) reported none. The rarity of this function was underlined by one respondent who described their own clinic's model in terms that explicitly framed it as exceptional: “*Social work is usually the lead and coordinating function in establishing work capacity and maintains close contact with the DI throughout the assessment and integration process. This is possible because [our clinic] has—unfortunately the unique possibility of—offering outpatient social counseling that can proactively accompany our patients through the DI process beyond the inpatient stay, acting as an interface between medicine and law*”*.* The framing of this model as “unfortunately unique” speaks directly to the structural gap that the majority of clinics cannot fill.

Vocational counseling was absent in eight clinics (73%), and only two had dedicated vocational counselors with formal qualifications. Where vocational counseling was offered by social work staff, it was described as focused on “*what is qualitatively and quantitatively still possible given the health limitations*”—reflecting the pragmatic and constrained scope of what counseling means in a rehabilitation context.

#### 3.1.3 Employer and insurer contact

Nine clinics (82%) contacted employers on behalf of patients, primarily to maintain the employment relationship, clarify functional status, and explore gradual return options. All clinics contacted the DI and/or accident insurance; health insurance contact was limited and mainly concerned neuropsychological follow-up. DI was contacted in most cases by six clinics and rarely by five. The nature of this contact varied considerably: some clinics described a proactive approach—“*Integration measures are initiated, and we try*—*through proactive contact*—*to shorten the waiting time for them; we proactively send the discharge report and provide the most accurate possible information about current work capacity and prognosis*”—while others reported limited contact constrained by the stage of the DI process: “*Rare contact [with the DI], as it is often the patient's first registration [with disability insurance]*.” This reflects a broader timing problem: when patients are first admitted to neurorehabilitation, they have often not yet engaged with the DI system, limiting what coordinated planning is possible during the inpatient stay.

Discharge marked a critical transition point for people with ABI. Nine clinics (82%) routinely referred patients to external vocational professionals; the remaining two did so case-dependently. No clinic reported never referring externally. Named external partners included specialized provider of outpatient neurorehabilitation, integrating RTW measures such as Rehapunkt, ZBA Luzern, Rehaklinik Bellion's vocational integration unit, contacts with the patient organization FRAGILE Suisse and Pro Infirmis and independent neuropsychological practices, and job coaches.

### 3.2 Survey of cantonal disability insurance offices

Fourteen out of the 20 invited DI offices located in the German-speaking part of Switzerland participated in the survey (response rate: 70%). As the DI office of the canton of Berne was represented by 15 staff members, individual responses were aggregated to a single office-level unit using modal responses. This resulted in a total of 14 office-level units included in the analysis. Respondents held roles such as case manager, vocational integration specialist, and vocational counselor.

#### 3.2.1 Service delivery structure

During the early intervention phase, most measures were predominantly delivered by external providers. Training courses were outsourced by 79% of DI offices, socio-vocational rehabilitation measures by 64%, and occupational measures by 71%. Workplace adaptations and technical aids were most commonly organized on a case-dependent basis (57% each). Counseling and support also followed a predominantly case-dependent approach (50%). Vocational counseling was the only early intervention measure without a clearly dominant delivery model, with responses distributed relatively evenly across all four delivery categories (Table 2).

**Table 2:** Service delivery structure for vocational measures, with service delivery type decided by disability insurance (DI): providers across cantonal DI offices (*N* = 14).

Measure	DI staff *n* (%)	External providers *n* (%)	Case-dependent (IV or external) *n* (%)	Shared (IV + external) *n* (%)	Don't know *n* (%)
(A) Early intervention phase (*N* = 14)					
Workplace adaptations	—	3 (21.4%)	8 (57.1%)	3 (21.4%)	—
Workplace aids assessment	1 (7.1%)	4 (28.6%)	8 (57.1%)	1 (7.1%)	—
Training courses	3 (21.4%)	11 (78.6%)	—	—	—
Job placement/job retention	1 (7.1%)	4 (28.6%)	7 (50.0%)	2 (14.3%)	—
Vocational counseling	5 (35.7%)	2 (14.3%)	3 (21.4%)	4 (28.6%)	—
Socio-vocational rehabilitation	2 (14.3%)	9 (64.3%)	2 (14.3%)	—	1 (7.1%)
Occupational measures	1 (7.1%)	10 (71.4%)	2 (14.3%)	—	1 (7.1%)
Counseling and support	3 (21.4%)	2 (14.3%)	7 (50.0%)	1 (7.1%)	1 (7.1%)
(B) Vocational integration phase (*N* = 14)					
Case management (Fallführung)	5 (35.7%)	—	5 (35.7%)	4 (28.6%)	—
Vocational-medical work capacity assess.	—	13 (92.9%)	1 (7.1%)	—	—
Counseling and support	1 (7.1%)	2 (14.3%)	8 (57.1%)	3 (21.4%)	—
Coaching	—	10 (71.4%)	3 (21.4%)	1 (7.1%)	—
Placement search (Suche Einsatzplatz)	—	7 (50.0%)	5 (35.7%)	2 (14.3%)	—
Socio-vocational rehabilitation: build-up training	—	13 (92.9%)	—	1 (7.1%)	—
Socio-vocational rehabilitation: work training	—	13 (92.9%)	—	1 (7.1%)	—
Occupational bridging (Zeitüberbrückung)	—	13 (92.9%)	—	1 (7.1%)	—
Vocational counseling (advisory sessions)	3 (21.4%)	2 (14.3%)	4 (28.6%)	3 (21.4%)	2 (14.3%)
In-depth vocational direction assessment	—	7 (50.0%)	3 (21.4%)	3 (21.4%)	1 (7.1%)
Formal/other vocational training	—	9 (64.3%)	1 (7.1%)	—	4 (28.6%)
Job placement/job search (Stellensuche)	—	5 (35.7%)	7 (50.0%)	2 (14.3%)	—
Workplace retention (Arbeitsplatzerhalt)	2 (14.3%)	2 (14.3%)	8 (57.1%)	2 (14.3%)	—
Trial employment (Arbeitsversuch)	2 (14.3%)	5 (35.7%)	4 (28.6%)	3 (21.4%)	—
Personnel leasing	—	5 (35.7%)	1 (7.1%)	—	8 (57.1%)

*N*  = number of DI offices. “—” indicates zero responses. Percentages are based on *N* = 14 offices. IV staff = measure delivered directly by disability insurance staff. External providers = external institutions or professionals contracted by the DI. Case-dependent = delivery varies by case. Shared = DI staff and external providers collaborate. Formal/other vocational training: Formal retraining and other training showed identical delivery patterns and are presented as a single row.

In the vocational integration phase, reliance on external providers increased further. Vocational-medical work capacity assessments were outsourced by 93% of DI offices, as were socio-vocational rehabilitation measures (both work-training and build-up measures) and temporary bridging employment (93% each). Coaching services were delivered by external providers in 71% of offices. Case management showed no dominant delivery mode: 36% of offices managed it internally through DI staff, 36% applied case-dependent arrangements, and 29% reported shared responsibility. Measures aimed at workplace retention were most often addressed through case-dependent arrangements (57%).

Decisions to involve external providers were described as driven primarily by available resources and the severity of health impairment, rather than by diagnosis-specific criteria. One respondent explained: “*It depends very much on the resources available at the DI office and on the severity of the health impairment. Where substantially more intensive support is required, an external provider is the primary recourse.*” Regarding socio-vocational training, another respondent emphasized that “*the first priority is always a placement in the open labour market with support from a specialized coach*,” while *noting that “there are no specialized institutions for people with a brain injury*” available for this type of training.

#### 3.2.2 ABI-specific provision and regional availability

Named external providers varied considerably across cantons. Specialist neuro-vocational centers—most prominently ZBA Luzern, Rehaklinik Bellikon, and Neuro Job Coaching—were cited repeatedly across multiple cantons; offices in the Bern area referenced a broader network of general providers (Rehapunkt, GEWA, BAND, BEWO, Avantos). Several offices noted the complete absence of specialist providers in their region: “*There is hardly any ABI-specific provision available. We therefore often fall back on the existing general job coaching institutions*” (Zürich); “*There is no institution in the canton of Bern that specializes in people with brain injuries and offers medical assessments*” (Bern); “*In peripheral regions, support from employers in the open labour market is indispensable*” (Graubünden). One respondent listed their regular external partners as “*the same providers we work with for other diagnoses*”. Desired providers also surfaced as unavailable due to financial and geographic barriers: institutions that “*are not satisfied with the DI reimbursement rates*” or “*do not cover our region*” (Bern).

#### 3.2.3 Perceived effectiveness and frequency of use

Coaching was rated as the most effective intervention, with 93% of DI offices rating it as “very effective.” Case management (86%), vocational-medical assessment (79%), counseling and support (79%), and trial employment (79%) were also rated very effective by a large majority of offices. Neither form of socio-vocational rehabilitation received any “ineffective” ratings. In contrast, training courses were rated as ineffective by 50% of offices, making them the most negatively assessed early-intervention measure. Temporary bridging work and personnel leasing were predominantly rated as “don't know” (71% and 86%, respectively), indicating very limited practical experience with these measures in the ABI context. Vocational counseling and retraining were each rated “don't know” by 29%–36% of offices and received “ineffective” ratings from 14% to 29%, reflecting uncertainty regarding their applicability for people with ABI (Table 3).

**Table 3:** Perceived effectiveness and frequency of use of vocational measures reported by disability insurance office staff (*N* = 14 offices).

Measure	Perceived effectiveness (*N* = 14)				Frequency of use (*N* = 14)			
	Ineffective *n* (%)	Quite effective *n* (%)	Very effective *n* (%)	Don't know *n* (%)	In most cases *n* (%)	≈Half of cases *n* (%)	Rarely/never *n* (%)	Don't know *n* (%)
(A) Early intervention phase								
Workplace adaptations	3 (21%)	6 (43%)	3 (21%)	2 (14%)	1 (7%)	5 (36%)	7 (50%)	1 (7%)
Workplace aids assessment	4 (29%)	4 (29%)	3 (21%)	3 (21%)	1 (7%)	4 (29%)	7 (50%)	2 (14%)
Training courses	7 (50%)	5 (36%)	—	2 (14%)	—	3 (21%)	8 (57%)	3 (21%)
Job placement/job retention	2 (14%)	5 (36%)	7 (50%)	—	8 (57%)	3 (21%)	2 (14%)	1 (7%)
Vocational counseling	4 (29%)	5 (36%)	1 (7%)	4 (29%)	2 (14%)	—	10 (71%)	2 (14%)
Socio-voc. rehabilitation	—	7 (50%)	6 (43%)	1 (7%)	3 (21%)	4 (29%)	4 (29%)	3 (21%)
Occupational measures	2 (14%)	5 (36%)	2 (14%)	5 (36%)	2 (14%)	1 (7%)	8 (57%)	3 (21%)
Counseling and support	—	6 (43%)	7 (50%)	1 (7%)	6 (43%)	5 (36%)	2 (14%)	1 (7%)
(B) Vocational integration phase								
Case management	—	1 (7%)	12 (86%)	1 (7%)	12 (86%)	—	—	2 (14%)
Work capacity assessment	—	2 (14%)	11 (79%)	1 (7%)	6 (43%)	4 (29%)	1 (7%)	3 (21%)
Counseling and support	—	2 (14%)	11 (79%)	1 (7%)	9 (64%)	2 (14%)	1 (7%)	2 (14%)
Coaching	—	—	13 (93%)	1 (7%)	6 (43%)	6 (43%)	—	2 (14%)
Placement search	—	4 (29%)	6 (43%)	4 (29%)	2 (14%)	6 (43%)	3 (21%)	3 (21%)
Socio-voc. rehab.: build-up training	—	5 (36%)	8 (57%)	1 (7%)	8 (57%)	3 (21%)	—	3 (21%)
Socio-voc. rehab.: work training	—	6 (43%)	7 (50%)	1 (7%)	5 (36%)	5 (36%)	—	4 (29%)
Occupational bridging	1 (7%)	1 (7%)	2 (14%)	10 (71%)	—	—	8 (57%)	6 (43%)
Vocational counseling	2 (14%)	3 (21%)	5 (36%)	4 (29%)	2 (14%)	1 (7%)	5 (36%)	6 (43%)
Retraining (Umschulung)	2 (14%)	2 (14%)	5 (36%)	5 (36%)	1 (7%)	2 (14%)	4 (29%)	7 (50%)
Job placement/job search	—	4 (29%)	9 (64%)	1 (7%)	4 (29%)	5 (36%)	1 (7%)	4 (29%)
Workplace retention	—	3 (21%)	9 (64%)	2 (14%)	6 (43%)	6 (43%)	—	2 (14%)
Trial employment	—	2 (14%)	11 (79%)	1 (7%)	3 (21%)	7 (50%)	1 (7%)	3 (21%)
Personnel leasing	—	1 (7%)	1 (7%)	12 (86%)	—	—	4 (29%)	10 (71%)

*N*  = number of DI offices. “—” indicates zero responses. Percentages are rounded to the nearest whole number.

Case management was the most consistently applied measure, reported as being used in most cases by 86% of offices. Counseling and support (64% most cases) and socio-vocational rehabilitation build-up training (57%) were also applied frequently. Coaching was used in most cases by 43% of offices and in about half of cases by another 43%. In contrast, vocational counseling and retraining were reported as rarely or never applied in the ABI context by 36%–50% of offices, accompanied by high “don't know” response rates (43%–50%). The near absence of retraining was reflected in explicit uncertainty, with one respondent noting that “*it is not known whether in any case the capacity for formal training was achieved following a brain injury*”. Regarding vocational counseling, one respondent reported that it was “*offered very rarely*” and that “*there are no external specialists available*” locally.

#### 3.2.4 Specialized expertise

Only two of 14 offices (14%) reported having staff with specific ABI expertise; even in offices where such expertise existed, 71% indicated that new ABI cases were not systematically assigned to these specialists. ABI-specific staff training was offered in 50% of offices, compared with 93% offering training related to mental health conditions, pointing to a structural deficit in ABI-specific expertise within the DI system.

### 3.3 Key features of an optimized, lifelong RTW service pathway

Based on previously gathered information and results of institutional surveys, we discussed and mapped the current RTW service pathway for people with ABI with our project partners. We have also identified gaps in the service provision for people with ABI which should be addressed to improve their return to work. We have grouped these gaps into four main themes: information, financing, communication and coordination. Topics belonging to the themes are not mutually exclusive but interconnected and partly overlapping (see Table 4). Findings indicate that an optimized service pathway, based on a 360° view that takes into account physical, cognitive, emotional needs, social situation, private and professional environment, and legal requirements, not only improves system efficiency but also reduces the personal and administrative burden placed on people with ABI, thereby supporting long-term work participation, work identity, and coping processes.

**Table 4:** Gaps in the current service provision presented by themes and related topics.

Theme	Topics
Information	What is ABI? Physical, cognitive, emotional, and behavioral effects
	Diagnosis and prognosis
	Medical measures and therapies
	Vocational rehabilitation—vocational measures
	Setting transitions
	Registration at the Disability Insurance Office
	Peer support
	Living with a brain injury—everyday life and work
Communication	Contacting the employer
	Setting transitions
	Registration with the Disability Insurance Office
	Support in finding a new job with a brain injury
	Peer support
Coordination	Setting transitions
	Registration with the Disability Insurance Office
	Financial issues
Financing	Financial issues
	Registration at the Disability Insurance Office
	Social benefits/Services and aids
	Financial benefits/continued salary payments
	Support in finding a new job with a brain injury
	Living with a brain injury—everyday life and work

Topics may appear under multiple themes, reflecting their interconnected role across information, communication, coordination, and financing processes within RTW service pathways.

**Figure 2:**
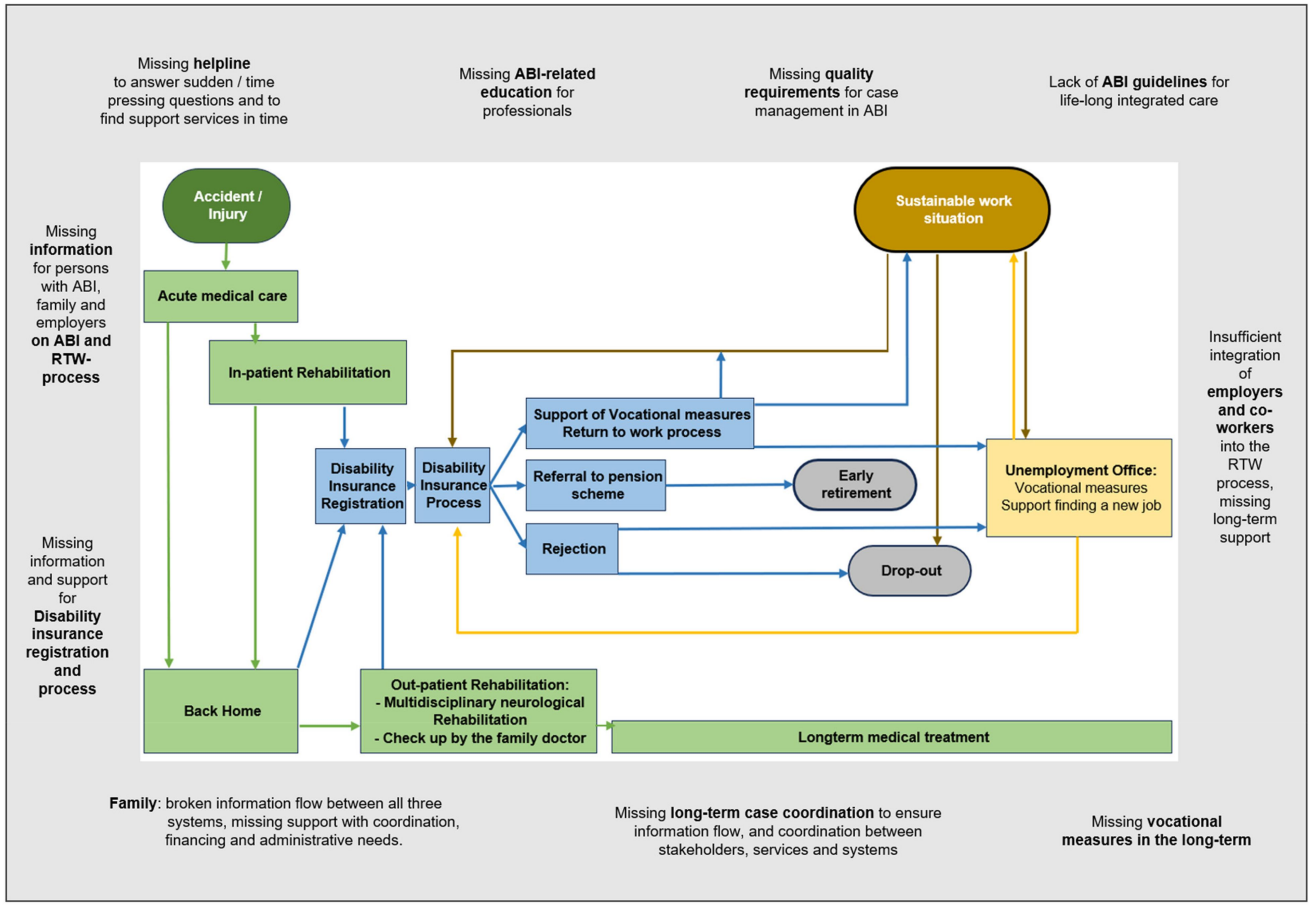
Schematic overview of the current return-to-work (RTW) service pathway, illustrating key interactions, transition points, coordination requirements, and existing service gaps.

**Figure 3:**
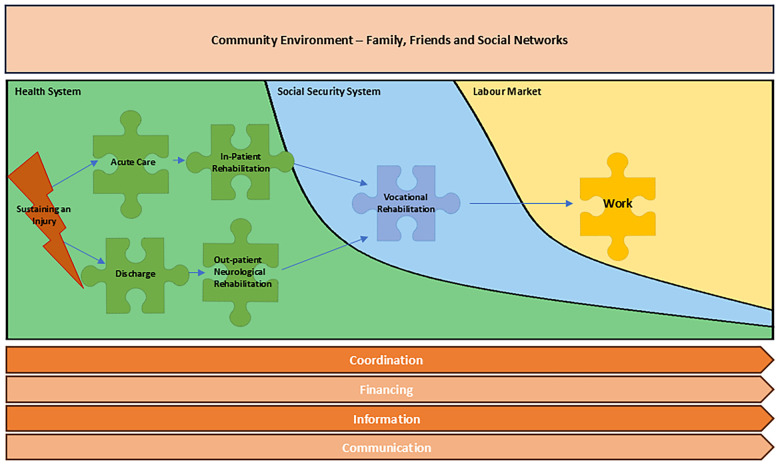
Schematic overview of key elements of an optimal, lifelong RTW service pathway across sectors. The three background colors represent the three institutional systems through which people with ABI move across their life course: green = Health System; blue = Social Security System; yellow = Labour Market. Gradual zone boundaries reflect cross-sectoral overlap and the need for continuity of care—individuals may be simultaneously engaged with more than one system, and transitions do not follow a fixed timeline. The Salmon banner (Community Environment) spans the full pathway, indicating that social environment is a constant contextual factor across all phases. Puzzle pieces represent distinct service components within each sector; their interlocking form conveys that effective RTW depends on the fit and coordination between adjacent services rather than any single component in isolation. The four orange banners (Coordination, Financing, Information, Communication) represent key elements of an optimal service pathway that must be actively maintained across all phases.

Simplified version of the current service RTW pathway is depicted in Figure 2 the crucial aspects of an optimal service pathway in Figure 3. While acknowledging the complexity of interactions between services and actors, the optimized pathway places the participation goal “sustainable work ability” at the centre and focuses on the main aspects needed for a high quality lifelong integrated service pathway “sustainable work ability after brain injury”.

### 3.3.1 Information

Stakeholders from all data sources identified missing or conflicting information as a major barrier to service utilization. One of the biggest challenges for people with ABI was the lack of information about the different steps in their rehabilitation and professional reintegration process and the transitions between them. They also reported incomplete instructions on administrative procedures, and legal requirements during the recovery process. In addition, both those affected and their relatives often did not know who to turn to with problems or questions.

Furthermore, a lack of knowledge and understanding among professionals, employers and colleagues about possible physical, cognitive and emotional limitations hindered people with ABI in returning to work and in finding long-term, sustainable employment.

People with ABI and their families need easy access to up-to-date information about ABI, its consequences and support services throughout their lives. Professionals need special education on ABI to provide information to people with ABI, employers and other stakeholders as pointed out by the patient.

Participants across all three stakeholder groups gave concrete expressions to these information gaps.

“I was discharged from rehabilitation without knowing that Fragile even exists. There was no brochure, nothing lying around. These cogs that should carry you out from the institution back home—they all have to mesh, otherwise nothing works” (Person with ABI).

“When I sat in the course on acquired brain injury and went through those materials, I asked myself: why do I have to register for a course like this to get this information? Everyone warns you about chronic fatigue—but what it actually means and how to handle it, you are left to work out yourself. I came out of that course thinking this is something everyone should know when they leave rehabilitation” (Person with ABI).

“DI staff simply do not know enough specifically about these clinical presentations. People make enormous efforts to conceal that they are running beyond their limit. Only when it is completely impossible any longer does a breakdown occur. I believe more trained staff are needed at the DI” (Health professional).

“If support were to go in that direction, it would mean explaining these clinical pictures and symptoms accessibly—background materials, simply explained, that one could hand to an employer or supervisor who finds themselves in this situation” (Employer of person with ABI).

### 3.3.2 Coordination

Fragmentation of services and lack of case management across the working life were mentioned as main obstacles in the service pathway. Good information flow between all stakeholders as well as transparency of processes, measures, and support options are needed to bridge different settings and link crucial stakeholders. One of the solutions that emerged from the gathered data would be life-long case management provided by central organization with knowledge of ABI, on medical, social and societal milestones and on legal procedures. Such an organization should provide lifelong support for those affected, helping them to deal with and coordinate all the stakeholders involved. In addition, guidelines for ABI that include medical, rehabilitative, social, vocational and financial aspects could ensure ABI-specific care, regardless of where the patient is treated.

Participants identified concrete coordination failures at multiple transition points across the service pathway.

“You fall completely out of working life, from one day to the next. Now come the first problems: the unemployment office, the disability insurance. These are obstacles that are actually supposed to help you. That is where a case manager belongs—someone who is there from the beginning and strengthens your back, who knows what rights you have and who also gives emotional support. That is what you need” (Person with ABI).

“Once a measure is running, there is someone in charge. But what happens afterwards varies enormously. Nobody feels responsible, because it is also not funded. Sometimes good employers have case managers who take care of it. But if they are not there, it is difficult” (Health professional).

“I had a concrete example in mind: a Spanish chef, formerly head cook in a canteen of 200 people. After his brain injury he was reintegrated slowly, and discussions were held both with the kitchen manager and the home director. After three to four years both had moved on. Then there was a phone call: they no longer understand me, they say I work too little. The new staff had simply not looked at what was in the files” (Health professional).

“What we also noticed was that the DI waited a very long time—until things were truly critical—before they felt they had to act” (Employer of person with ABI).

### 3.3.3 Communication

Communication was identified as another crucial aspect in the service pathway. Transparent and open communication between different stakeholders was mentioned as a strong facilitator to ease RTW and rehabilitation related processes across service continuum. As the employer is a crucial stakeholder in RTW process it has been suggested that contact should be established as soon as possible to prevent mistrust and logistic difficulties. On the other hand, employee is not obliged to share the reason for the work absence and the diagnosis apart from work-related limitations. Lack of knowledge about the employee's health condition make it sometimes difficult for employers and supervisors to support RTW and work adjustments. Lack of such knowledge may also diminish possible support of co-workers.

Communication was also mentioned as important in contact with benefits providers—health, accident and disability insurances.

People with ABI, professionals involved in their rehabilitation and vocational integration, as well as colleagues and employers in their daily work should be aware of the importance of communication and know how to contribute to good communication.

Stakeholders highlighted the particular communication challenges posed by the invisible nature of ABI, both within the workplace and toward benefit providers.

“I have been concealing it for 26 months at work and in the 26 months I have been at my current employer nobody has noticed. At the last place, once I said what I had, I immediately received my notice. So I am afraid that if I say something, the trust in my abilities will disappear” (Person with ABI).

“The message from me—everything is fine again—that message never comes. And I sense in the people around me that they would so dearly like to hear it. But it simply does not come, because it just is not so. After about a year the understanding has evaporated. The expectation becomes: you have a 50 per cent contract, now deliver” (Person with ABI).

“Open and transparent communication on all sides is what I advocate—dealing honestly with both strengths and weaknesses. It really has to be on the table. Otherwise, the employer quickly feels misled—feels they have bought a pig in a poke—and then it does not work out and the relationship of trust collapses” (Health professional).

“It needs to be made transparent by those living with the brain injury—what exactly their challenges are. This can then lead to positive awareness-raising among other team members. But this requires that the person has a reflective relationship with their own injury” (Employer of person with ABI).

### 3.3.4 Financing

Financial barriers emerged as a structurally embedded cross-cutting issue along the RTW service pathway. People with brain injuries face two main financial challenges in returning to work: maintaining income during sick leave and funding medical, rehabilitative, and vocational measures.

In Switzerland, wage continuation during sick leave is covered by employers or daily benefits insurance for illness, and by accident insurance for accidents. Payment terms depend on employment contracts and legal regulations, which vary by employment length, industry, and canton. DI covers wage payments during occupational measures it finances and can provide a pension after one year if a permanent disability is expected.

Since wage payments or pensions are often significantly lower than pre-injury salaries and may not cover the full illness period, many face financial hardship. Affected persons often struggle to manage financial matters independently. While social services can assist with financial organization, individuals or their families must actively seek help. These services typically offer only short-term support.

Medical treatment costs are covered by health or accident insurance, but a lack of ABI-specific guidelines leads to reduced funding for emergency, acute, and rehabilitation stays. This can result in incomplete ABI diagnoses and inadequate follow-up care. Inpatient rehabilitation is increasingly constrained in length of stay, social support, and discharge planning, leading to disorganized outpatient care and delayed support for professional reintegration, which may result in job loss.

Vocational measures are financed by DI, but timely registration—ideally supported by rehabilitation professionals—is crucial. Approval depends on meeting baseline performance levels, which persons with ABI often fail to achieve. Consequently, they may be placed on a pension, losing access to vocational integration and job placement support.

Long-term support, such as job coaching for employees and employers, is time-limited. Although DI can still be contacted after integration, long waiting times and administrative hurdles discourage applications for support.

To ensure integrated care that encompasses medical, rehabilitative, and integrative measures, all funding agencies should be involved at an early stage of the recovery and integration process. Financial responsibilities should be clearly delineated and transitions planned in advance, with due consideration of the individual's life situation throughout.

The financial consequences of these systemic gaps were acutely felt by individuals with ABI, their families, and employers.

“In Switzerland, if I say it that way—thank goodness someone ran me over, and I did not have a brain tumor or a hemorrhage. That is what it comes down to in our system. At least you have the advantage that you had an accident, they tell me—and I already find that tragic” (Person with ABI).

“Those of us who have to hold so many jobs just to make ends up a little—that seems to belong with us. I can still work 40 per cent in permanent employment and alongside that I have my own studio. Only two jobs” (Person with ABI).

“What I hear more and more is that daily allowance insurers are applying additional pressure—effectively wanting people to take up a different activity even while certified sick. Then they do not have to keep paying. This is a new trend, and it has a very negative effect on the reintegration process” (Health professional).

“The insurer wanted him to appear as capable as possible so that no insurance benefits would be needed. If I had presented everything as being fine again, I would have harmed him in the long run” (Employer of person with ABI).

## 4 Discussion

Taken together, the results provide a detailed, system-level analysis of current RTW service pathways for people with ABI in Switzerland. They reveal how persistent fragmentation across healthcare, social security, vocational integration, and employment systems undermines sustainable work participation over time. By integrating institutional surveys, prior qualitative research, and stakeholder co-production within an IKT framework, the study moves beyond identifying isolated barriers and offers a longitudinal, cross-sectorial service pathway perspective.

Many of the barriers identified in this study—gaps in information, coordination, communication, and financing—are consistent with previous international RTW and ABI literature. In particular, out findings align with Lefebvre et al. (21), and related studies that describe deficits in information provision, coordination, continuity of support, and financial protection across the rehabilitation and community reintegration process. Similar themes have also been described in studies from Australia, Canada, the Netherlands, Denmark, and the United States, suggesting that fragmentation is a common feature of RTW pathways for people with ABI across highly differentiated systems (46–51). The novel contribution of the present study lies not in identifying these barriers *per se*, but in showing how they interact dynamically across systems and accumulate at critical transition points along the work life.

A key novel contribution of this study lies in conceptualizing RTW after ABI as a cross-sectoral, lifelong service pathway rather than as a discrete rehabilitation outcome or a series of isolated interventions. Mapping the “as-is” pathway made visible critical transition points—such as discharge from inpatient rehabilitation, initial contact with disability insurance, and post-integration employment maintenance. At these points, responsibilities often become unclear or discontinuous, effectively shifting coordination tasks onto individuals with ABI and their families—despite cognitive, emotional, and fatigue-related impairments that limit their capacity to navigate complex systems independently (52).

Based on these findings, proposed optimizations address multiple levels: micro, mezzo, macro. Key issues consider coordination of services within and across systems, keeping employers on board, providing easily accessible long-term support for people with ABI, and raising knowledge and awareness among health professionals (GPs, insurance and vocational rehabilitation specialists). Among the four cross-cutting themes identified, coordination across settings and sectors over time emerges as particularly central.

In practice, only a small number of “lighthouse projects” in Switzerland offer integrated care spanning medical rehabilitation, vocational rehabilitation and sustainable work integration beyond system boundaries. These initiatives typically rely on the personal commitment of individual professionals and the voluntary cooperation of actors across different sectors, united by the shared goal of providing optimal support for the individuals returning to work. However, these initiatives remain exceptions rather than standard practice. As they are not embedded in standardized or nationwide RTW service pathways, access to integrated support remains uneven, and many individuals do not receive optimal follow-up despite the existence of individual services.

Closely related to coordination is the flow of information. Our findings suggest that information gaps arise primarily as a consequence of inadequate coordination mechanism. Without established and binding coordination pathways across systems and settings, information is frequently fragmented or lost. People with ABI as well as health professionals and employers consistently express a need for clearer and more accessible information on the consequences of ABI, RTW options, and available support measures. Strengthening coordination structures with reliable information flows could substantially improve collaboration across stakeholders, including service providers, insurances representatives and employers.

Financing represents another major structural challenge. Multiple stakeholders are involved in the provision of medical, rehabilitative, and vocational measures, with costs divided between payers from the health care and disability insurance systems. However, founding responsibilities may vary by canton, cause of injury and the insurance status, creating uncertainty and delays. Multi-stage approval processes further contribute to confusion, and services perceived as essential—such as legal advice—are often not reimbursed. Even professionals involved in insurance and vocational rehabilitation may provide incomplete or inconsistent information on funding responsibilities, hindering timely access to support.

The available evidence suggests that committed employers play a crucial role in successful RTW after disability. However, employers' ability to understand and implement appropriate workplace adjustments may be limited by the legal context in Switzerland. Under Swiss law, employees are not obliged to disclose a medical diagnosis or its underlying causes. Instead, they are required only to inform the employer about their current ability to work and any relevant functional limitation. This legal framework can complicate communication, particularly in cases of ABI, where impairments may be invisible or fluctuate over time.

A trusting employee-employer relationship, supported by professionals such as job coaches or vocational integration specialists, are therefore crucial for facilitating transparent communication and sustainable reintegration. In many cases, transparent factual communication about functional capacities and support needs is a prerequisite for employers and colleagues to develop sustained understanding and realistic expectations following RTW. In addition, employers expressed a need for greater administrative and financial support to adequately prepare for and manage the reintegration process.

Recent legislative changes enable DI to intervene at an early stage through early intervention services. Measures aimed at job retention or supporting RTW can now be financed promptly, even before completion of a comprehensive eligibility assessment. These measures include, among others, communication with employers and coordination of reintegration measures across settings. This development is promising, as it addresses fragmentation and financing challenges from an institutional perspective. However, its practical impact varies depending on implementation practices and awareness among professionals and affected individuals. In particular, specialized job coaching, supported by improved information flow and coordination between professionals and employers and integration measures beyond the boundaries of the health and social system, appears to hold significant potential for strengthening sustainable RTW pathways.

Nevertheless, people with less visible limitations and those with more severe disabilities continue to face major challenges, as they are often not entitled to vocational reintegration support from DI. In addition, many affected individuals are unaware that DI has a legal mandate to prioritize vocational reintegration before granting a disability pension. This gap in awareness appears especially pronounced among individuals who have not undergone inpatient rehabilitation and who must navigate the reintegration process largely on their own. From a system perspective, these findings point to structural and information barriers that may limit access to reintegration measures, underscore the need for clearer communication and earlier outreach rather than placing responsibilities solely on affected individuals.

Bridging the gaps between sectors is critical for sustainable employment, as a RTW service pathway aimed at workforce participation includes not only healthcare services, but also vocational integration, social assistance, and funding mechanism (53). To support sustainable work participation among people with ABI, services cannot be limited to medical care alone; they must also encompass social and psychological support tailored to the needs of each individual and their family within their living environment. Accordingly, bridging the gap between medical care or rehabilitation and community integration requires a shift from purely medical perspective toward a more comprehensive biopsychosocial approach (54). A particularly critical point within this pathway is the transition from first rehabilitation to community-based integration. At this stage, individuals with ABI are often left to manage ongoing challenges largely on their own or with family support. In many cases, a timely and well-coordinated transfer to outpatient specialists who are connected to vocational and social services could enable earlier initiation of appropriate work-related measures and help prevent deterioration of psychological wellbeing.

Although, no single “gold standard” exists, available evidence suggests that case management is particularly effective in complex cases (55). Within this model, a case manager develops an individualized intervention plan, identifies and coordinates required services, and supports the person with ABI throughout the rehabilitation and RTW process. Given the complexity and heterogeneity of ABI, comprehensive assessment of cognitive, behavioral, and functional consequences is essential, underscoring the need for skilled and experienced professionals to design and implement effective re-entry programs.

### 4.1 Strengths and limitations

A key strength of this study lies in its stepwise, pathway-oriented design, the integration of multiple empirical data sources, and the application of an IKT approach.

Unlike many previous studies, this work combines findings from institutional surveys, secondary qualitative analyses, and the practical experience of knowledge users, thereby providing a 360° system-level perspective on RTW service delivery, ranging from acute care to long-term employment retention.

Close collaboration between researchers and experts from different disciplines enabled the co-production of knowledge throughout the research process. This collaborative approach is expected to enhance the relevance, feasibility, and practical applicability of the findings and to increase the likelihood that the results will inform both practice and policy.

However, several limitations should be considered when interpreting the findings. Although the project team was purposively composed to cover key perspectives across the RTW service pathway, this approach—consistent with the IKT framework—may have influenced the resulting pathway and interpretations. In addition, the Swiss health and social security system is highly fragmented, and some aspects of service provision may not have been fully captured. In particular, data on outpatient services were less comprehensive than data on inpatient neurorehabilitation clinics. Information on outpatient care relies primarily on data from a previous project and from representatives of DI and may therefore be underrepresented.

In addition, the findings are specific to the Swiss context and are based primarily on data from the German-speaking part of Switzerland. While the legal frameworks governing DI and related payers are comparable nationwide, regional and cultural differences in service organization, professional practice, and labor-market dynamics may influence RTW processes. As a result, patterns of vocational reintegration observed in the German-speaking regions may not fully reflect those in the French- or Italian-speaking parts of Switzerland.

Finally, as the findings are specific to the Swiss context, they are characterized by strong cantonal governance and distinct insurance structures, which may limit direct transferability to more centralized health and social security systems.

### 4.2 Practical and policy implications

As a first step toward improving navigation within the current system, all parties involved—including people with ABI—need to be aware of the overarching factors that shape RTW outcomes, namely information, communication, coordination, and financing, and how these interact across the service pathway. Practical recommendations addressing these aspects at the micro and mezzo levels may lead to immediate improvements in practice by fostering better coordination and more efficient use of available resources. For example, people with ABI and their relatives should be provided with written, accessible information about the consequences of ABI and about available sources of support. At the mezzo level, improved education and training of health and insurance professionals could better equip them to guide through recovery, vocational reintegration, and long-term work participation after ABI. The use of case management should be considered on a case-by-case basis. Evidence from studies in Switzerland (19, 20) supported by earlier work by Cope (56) highlight the potential benefits of case management, including shorter inpatient stays and improved rehabilitation outcomes.

General practitioners (GPs) play a particularly important role, as they are often the first point of contact for a person with ABI. They should be able to recognize early signs of health deterioration, understand implications for work participation, and initiate appropriate social and occupational support measures. This role is especially critical for individuals who have sustained a mild injury and were not hospitalized, and who may otherwise receive little structured follow-up.

At the macro level, the issues identified in this study point to the need for policy-level changes. A system-wide financing framework oriented towards social and professional integration after ABI could better align the timing of work integration measures with individual recovery trajectories and enable long-term support where needed. In addition, national policies supporting the development of integrated data collection and electronic case record systems, enabling information sharing across stakeholders under appropriate data protection standards, could facilitate smoother transitions between settings and reduce information gaps across systems.

In many countries, data on service utilization are derived primarily from administrative records and provider payment documentation. The more fragmented a healthcare system is, the more challenging it becomes to link such data in order to describing service pathways comprehensively. International studies have identified several barriers, including fragmented data ownership, limited semantic comparability across databases, data protection concerns, and technical challenges associated with managing large volumes of data (57).

## 5 Conclusions

Return to work service pathways represent a promising approach to service optimization, as they enable systematic identification of structural gaps and potential intervention points across systems. This study identified key obstacles in the current service landscape for people with ABI and highlighted how these barriers interact over time within fragmented health, social security, and labour-market context. These findings suggest that improvements are needed both through short-term practice-oriented measures and through long-term policy and structural reforms.

By actively involving multiple stakeholders through an IKT approach, this study increases the likelihood that the proposed pathway-level insights and recommendations will be perceived as relevant, feasible and actionable. Engaging these stakeholders as agents of change may, in return, enhance the chances that recommendations are acknowledged and implemented in practice, ultimately contributing to more coordinated and sustainable return-to-work outcomes for people with ABI.

## Data Availability

The raw data supporting the conclusions of this article will be made available by the authors, without undue reservation.
